# High-throughput electron diffraction in the XtaLAB Synergy-ED

**DOI:** 10.1063/4.0000861

**Published:** 2025-10-27

**Authors:** Robert Bucker, Mathias Meyer, Michal Jasnowski, Mateusz Idzi, Jessica Burch

**Affiliations:** 1 Rigaku Europe SE, Neu-Isenburg, Neu-Isenburg, Germany; 2 Rigaku Oxford Diffraction, Wrocław, Wrocław, Poland; 3 Rigaku Americas, The Woodlands, TX, USA

## Abstract

Microcrystal electron diffraction (MicroED/3D ED), which solves atomic structures from crystals in the sub-micron size range using electron radiation, has evolved from an exploratory technique into a cutting-edge analytical method practiced in laboratories worldwide. However, viewing MicroED solely as an extension of single-crystal diffraction, traditionally performed with X-rays on smaller crystals, underestimates its capabilities. With thousands of crystallites available simultaneously on a sample grid and measurements completed within minutes, we can obtain a more comprehensive view of our specimen by collecting crystallographic data from dozens or even hundreds of crystals. This approach allows for the analysis of powders containing mixtures of compounds, different polymorphs, or even small traces of contaminants, including ab-initio solutions, even in cases too complex for powder diffraction or where only minute sample amounts are available. In this contribution, we will discuss how high-throughput workflows in the Rigaku XtaLAB Synergy-ED electron diffractometer enable various beyond-single-crystal investigations with a high degree of automation and ease of use.

